# Ontologically spiky: Behind trichome formation in lilies sepals

**DOI:** 10.1093/plphys/kiaf063

**Published:** 2025-03-04

**Authors:** Sebastián R Moreno

**Affiliations:** Assistant Features Editor, Plant Physiology, American Society of Plant Biologists; Sainsbury Laboratory, University of Cambridge, Bateman Street, Cambridge CB2 1LR, UK

**Figure 1.**
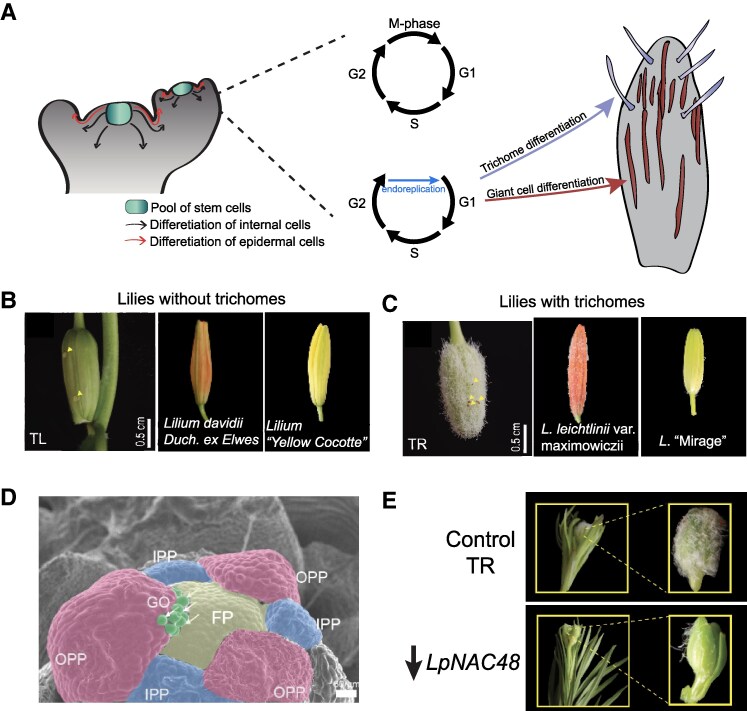
Trichomes as a model to understand cell differentiation in plants. **A)** Differentiation trajectories in plants are defined by the initial location of the mother stem cell. Differentiation trajectories could follow internal and epidermal lineages. Trichomes are defined by epidermal undifferentiated cells. **B)** TL and other lilies varieties without trichomes in their sepals. **C)** TR and other lilies varieties with trichomes in their sepals. **D)** Trichomes are initiated during sepals are elongating. GO, globular outgrowth; OPP, outer perianth primordium. **E)** LpNAC48 regulates trichome formation. B to E images were obtained from Xin et al. 2025.

During development, cells differentiate into a diverse array of types, each of them tailored to perform specific functions within the organism. In plants, stem cells transition into specialized cell types with specific cell functions, morphologies, and molecular landscapes (Fig. 1A). Cell fate progression behind any cell type is orchestrated by the division plane and the spatial position of the mother cell within the stem cell pool. Epidermal stem cells, for instance, give rise to various cell types such as pavement cells, stomatal guard cells, and trichomes (Kuan et al. 2022).

Trichomes, or shoot epidermal hairs, are found in most of aerial organs such as leaves, sepals, and stem (Xu et al. 2021). Trichomes, which can be either unicellular or multicellular, serve a variety of functions such as reducing transpiration and protecting plants from insect damage and UV light (Hülskamp 2004). Some epidermal cells stop the mitotic cycle and initiate endoreplication cycles that increase cell size through a differentiation process that ends with a variety of morphologies depending on the organ and species (Fig. 1A) (Schnittger and Hülskamp 2002). Based on morphology, trichomes are usually classified in branched or unbranched, glandular or glandless (Wang et al. 2021).

Previously, a natural variant of *Lilium pumilum* with trichome covered perianths (TR, trichome) was isolated, providing a model for understanding trichome formation in lilies (Fig. 1, B and C) (Xin et al. 2021). In this issue of *Plant Physiology*, Xin et al. 2025 delved into the underlying mechanisms of trichome formation in this TR variant and its trichome-less counterpart (TL).

The authors tracked trichome formation at different developmental stages, noticing that trichomes in the TR variant appeared during flower bud development, forming multicellular, branchless, and nonglandular trichomes (Fig. 1D). The authors also observed that the higher trichome density in the TR variant provided protection against aphids and UV-B radiation, revealing new insights about the role of trichomes in plant defense.

Leveraging previous studies that compared gene expression profiles in TR with those in TL variants (Xin et al. 2021), Xin and collaborators identified 7 potential key transcription factors (TF) with *LpNAC48* showing the highest expression in TR flower petals during early bud formation. In TL, the authors observed that *LpNAC48* was mostly expressed in leaves. The authors further validated the role of *LpNAC48* in lily trichome formation by silencing it in TR flower bulbs. Silencing *LpNAC48* in TR flower buds led to fewer trichomes (Fig. 1E).

The research then showed that LpNAC48 physically interacts with the TF LpBBX28, which is also upregulated in TR compared with TL. Notably, silencing LpBBX28 in TR bulbs leaded to reduction in the number of trichomes on the flower buds, confirming its role as a relevant regulator in trichome formation. In order to link the new regulatory network with previously known trichome-related genes, the authors confirmed that *LpGL3* was upregulated in the *LpNAC48*-overexpressing lines. Moreover, the authors showed that LpNAC48 and LpBBX28 synergistically bind to the *LpGL3L* promoter.

**Figure 2.**
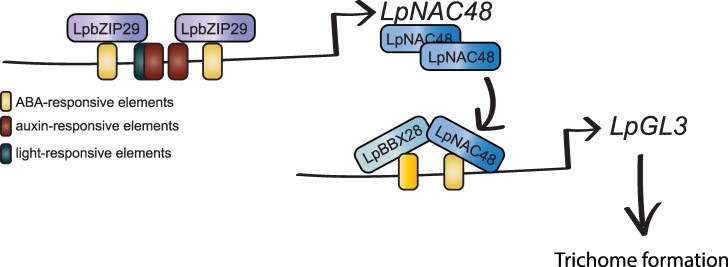
Proposed gene regulatory network behind trichome formation in lilies. LpbZIP29 TF binds to ABA-responsive elements to promote the upregulation of *LpNAC48*. Then, LpNAC48 binds to cis-regulatory elements in the promoter of LpGL3, a key player behind trichome formation.

Finally, to understand the role of LpNAC48 in causing the phenotypic differences between TR and TL varieties, the authors focused on the *LpNAC48* locus and identified a 259-bp difference in the NAC48 promoter of the 2 natural varieties and between other varieties with or without trichomes (Fig. 2). This promoter region contained elements related to abscisic acid response, light response, and auxin response. Leveraging previous transcriptomic assays, the authors found *LpbZIP29* with previously reported abscisic acid response function (Van Leene et al. 2016). Overexpressed *LpbZIP29* in TR and TL flower buds led to the upregulation of *LpNAC48* and *LpGL3L* in TR but not clear regulation in the TL varieties, which lack the 259-bp promoter fragment. Then, the authors confirmed the direct role binding of LpbZIP29 in the ABA-responsive elements of the promoter within the analyzed 2459 bp by different methods such as electrophoretic mobility shift assay, chromatin immunoprecipitation-reverse transcription quantitative PCR, and yeast one-hybrid. In addition, mutations in the binding sites also confirmed these motifs are relevant for the upregulation of *LpNAC48* in TR.

Thus, through meticulous and clean rationale set of experiments, the authors uncovered a novel regulatory network driving trichome formation in lilies (Fig. 2). Notably, they established a connection between ABA-responsive elements and trichome formation, suggesting a broader role for ABA in plant development and cell differentiation. Both pavement cells and trichomes arise through endoreplication process from undifferentiated epidermal cells, yet they exhibit markedly different cellular functions and morphologies. Thus, trichome formation is a good model to determine when cells commit to one differentiation pathway over the other. Future approaches, such as fluorescence-activated cell sorting single-cell RNA-seq tracking trichome formation could give us valuable insight behind this process. Overall, Xin and collaborators have significantly expanded our understanding of the mechanisms governing this distinctive cell type.
